# Dermal Duct Tumor: A Diagnostic Dilemma

**DOI:** 10.3390/dermatopathology9010007

**Published:** 2022-01-28

**Authors:** Austinn C. Miller, Susuana Adjei, Laurie A. Temiz, Pavandeep Gill, Alfredo Siller, Stephen K. Tyring

**Affiliations:** 1 Center for Clinical Studies, Webster, TX 77598, USA; sadjei@ccstexas.com (S.A.); laurietemiz@gmail.com (L.A.T.); asillermd@gmail.com (A.S.J.); styring@ccstexas.com (S.K.T.); 2 School of Medicine, Meharry Medical College, Nashville, TN 37208, USA; 3 Department of Pathology, Royal Jubilee Hospital, Victoria, BC V8R 1J8, Canada; pavandeep.gill@islandhealth.ca; 4 Department of Dermatology, The University of Texas Health Science Center, Houston, TX 77030, USA

**Keywords:** dermal duct tumor, DDT, poroma, poroid, hidradenoma

## Abstract

Poromas or poroid tumors are a group of rare, benign cutaneous neoplasms derived from the terminal eccrine or apocrine sweat gland duct. There are four poroma variants with overlapping features: dermal duct tumor (DDT), eccrine poroma, hidroacanthoma simplex, and poroid hidradenoma, of which DDT is the least common. Clinically, the variants have a nonspecific appearance and present as solitary dome-shaped papules, plaques, or nodules. They can be indistinguishable from each other and a multitude of differential diagnoses, necessitating a better understanding of the characteristics that make the diagnosis of poroid neoplasms. However, there remains a paucity of information on these lesions, especially DDTs, given their infrequent occurrence. Herein, we review the literature on DDTs with an emphasis on epidemiology, pathogenesis, clinical features, diagnosis, and management.

## 1. Introduction

Acrospiromas are a broad class of benign skin adnexal tumors of acrosyringial differentiation. A group of benign growths derived from cells of the terminal eccrine or apocrine sweat gland duct, known as poromas or poroid tumors, fall within this class [1]. There are several types of poromas: dermal duct tumors (DDT), eccrine poromas, hidroacanthoma simplex, and poroid hidradenomas [1]. Each is differentiated by histopathological features such as lineage (eccrine/apocrine) and location of the poroid cells in relation to the epidermis [2]. It is estimated that sweat gland tumors account for approximately 1% of all primary skin lesion cases, of which eccrine and apocrine poromas are believed to account for approximately 10% [3]. However, there remains a paucity of information on poromas, especially DDTs, given their infrequent occurrence.

## 2. Discussion

DDTs are predominantly located within the dermis and thus are also known as intradermal or dermal eccrine poromas [2]. They are composed of a mixture of three types of cells: poroid, cuticular, and clear cells [2]. These features are key in differentiating DDTs from other poroma variants. However, all of these variants have features that can overlap.

## 3. Epidemiology

While the exact incidence of poromas is unknown, several studies have revealed specific epidemiological data. One single center study reviewed approximately 1,741,379 epithelial skin tumor biopsies over 17 years and noted the occurrence of 101 poromas (0.0058%) [4]. Another single center study examining 18,653 biopsy specimens processed over 15 years revealed 25 poroid neoplasms (0.134% of all pathology) [5]. The presence of all poroma variants except DDT was noted in this particular study. Across 3 studies examining 675 cases of poroid neoplasms, solitary DDT was the rarest variant, occurring only 22 times (3.3%) [5,6,7].

Poromas typically occur in the elderly population (mean age 65.1–66.6) with a slight predilection for males (male to female ratio: 1.13–1.52) [4,6]. Long-term radiation exposure has been associated with poroma formation in patients with chronic radiation dermatitis [8]. Poroma eruptions have also occurred during pregnancy and after bone marrow transplantation [9,10]. In addition, poromas may occur in patients with underlying skin conditions, including hypohidrotic ectodermal dysplasia, Bowen’s disease, and nevus sebaceus [8].

## 4. Pathogenesis

**Figure 1:**
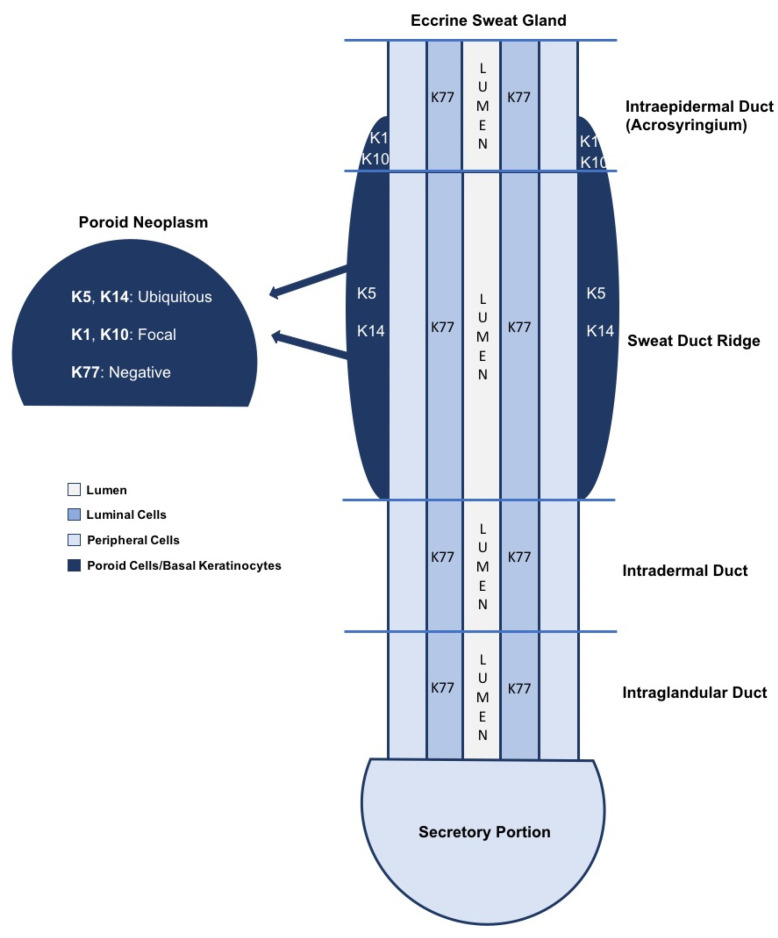
A simplified schematic of keratin expression in an eccrine sweat gland is shown [6,10]. The basal keratinocytes of the sweat duct ridge and lower intraepidermal acrosyringium are thought to give rise to poroid neoplasms based on the similarities in keratin expression [6,10]. K5 and K14 expression is ubiquitous throughout poromas, while K1 and K10 are found in focal aggregates. K77 is restricted to normal luminal cells embedded within the tumors.

The exact pathogenesis of poroid neoplasms is unknown, although immunohistochemical studies have provided clues (Figure 1). Based on the ubiquitous expression of keratin K5 and K14 in poroid cells, it has been postulated that poromas are periductal sweat gland tumors derived from basal keratinocytes of the sweat duct ridge and the lower acrosyringium [6,11]. As basal keratinocytes differentiate and advance to the upper acrosyringium, cells express keratins K1 and K10, which are found within cuticular cells. Further supporting the basal keratinocyte origin is the lack of luminal duct keratins (K77) [6]. Basal keratinocytes of the sweat duct ridge constitute an outermost third layer of the dermal bilayered ductal structure, merging with the adjacent epidermis [6]. This third layer has a highly variable length, which could account for the various forms of poroid neoplasms, according to the initial site of tumor induction [6,11].

More recently, YAP1 gene fusions have been implicated in the tumorigenesis of poroid neoplasms. The exact role of YAP1 in poromas has not been elucidated; however, results from 2 studies revealed certain fusions (*YAP1-NUTM1* or *YAP1-MAML2*) were present in 113/146 (77.4%) poromas [12,13]. However, only 3 DDTs were studied, with a 2/3 (66.7%) rate of expression. While more extensive studies are needed, its presence may serve as a diagnostic aid in uncertain cases.

## 5. Clinical Features

Clinically, poroma variants are typically indistinguishable. Moreover, poromas are often misdiagnosed as other skin neoplasms because their clinical presentations are nonspecific and variable [3]. In general, poromas present as solitary dome-shaped papules, plaques, or nodules [1]. They are usually slow growing and asymptomatic, although some patients may experience itching and pain [8]. Lesional color ranges from skin-toned to pink, red, white, or blue [8]. The surface may be smooth, verrucous, or ulcerated [14]. They may occur anywhere on the body, but are most commonly seen on acral, head, and neck surfaces [1,2]. Rarely, multiple poromas will develop, either in an acral or in a widespread distribution, a condition known as poromatosis [15].

**Figure 2:**
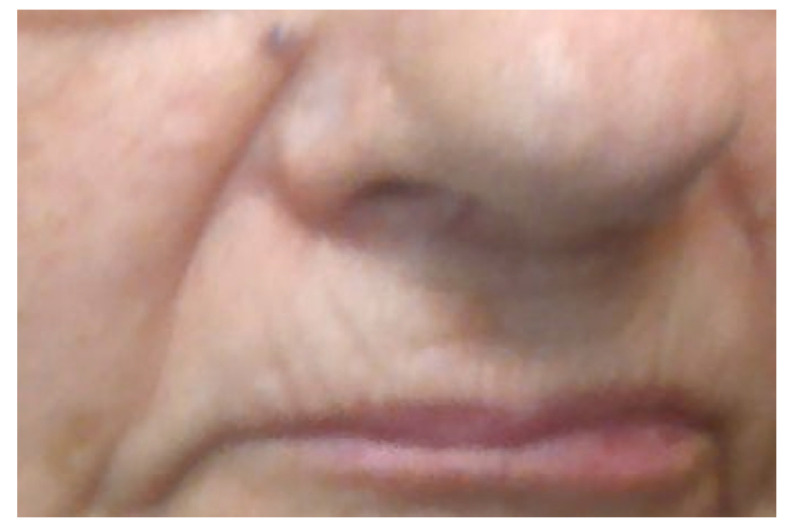
Dermal duct tumor: a small, purple-brown papule at the right superior nasolabial fold.

Battistella et al. reviewed 19 cases of DDTs and revealed that lesions were most commonly described as small dermal nodules (50%), infiltrative dermal papules (40%), or pedunculated tumors (10%) [6] (Figure 2). Color ranged from pink to erythematous (69%), sometimes pigmented (25%), and rarely bluish (6%). Epidermal surface changes were only noted in one case of combined hidradenoma simplex and DTT, in which the lesion was verrucous [6]. No ulceration was noted. Pain was mentioned in 1 case [6]. The trunk was the most common lesion site 39%, followed by the upper limbs (28%), lower limbs (28%), and head/neck (1%) [6]. The location of 1 lesion was unknown. Lesion size was <1 cm in 69% of cases [6].

## 6. Differential Diagnosis

Differential diagnosis of DDTs includes all poroma variants (eccrine poroma, hidroacanthoma simplex, and hidradenomas) and other sweat gland tumors (Table 1). Other differentials include porocarcinoma, basal cell carcinoma, pyogenic granulomas, acrochordons, verrucae, soft fibroma, hemangioma, pigmented nevus, seborrheic keratosis, trichilemmoma, melanoma, Kaposi sarcoma, and other adnexal tumors [1]. Dermatoscopy can be utilized to narrow down the differential, but confirmation relies on histopathology.

Of the 19 DDTs reviewed by Battistella et al., the clinical diagnosis was most often dermatofibroma (33%), followed by dermal melanocytic nevus (28%), melanoma in pigmented cases (22%), and benign dermal tumors (neurofibroma, leiomyoma) (17%) [6].

## 7. Diagnosis

### 7.1. Dermatoscopy

While there are no specific reports reviewing dermatoscopic findings in DDTs, certain vascular patterns have been noted to aid in diagnosis of poromas. Polymorphic, glomerular, linear-irregular, leaf- and flower-like, and looped or hairpin variants are commonly seen [8]. While these patterns may be seen with other conditions, the leaf- and flower-like pattern appears to be relatively unique to the poromas [27]. Additional dermatoscopic features included the presence of vascular blush secondary to the vasodilatation and high vascular volume of these tumors, as well as structureless areas and white interlacing cords [1].

The predictive value of white interlacing areas around vessels, yellow structureless areas, milky-red globules, poorly visualized vessels, and branched vessels with rounded endings has been studied [28]. The presence of any of these 5 features was associated with poroma with a sensitivity and specificity of 62.8% and 82.0%, respectively [28].

### 7.2. Histology

Ultimately, differentiation occurs via histopathology. The principal finding in all forms of poroma is a circumscribed proliferation of compact cuboidal keratinocytes with small non-palisading monomorphous nuclei with scant eosinophilic cytoplasm (poroid cells) and larger squamous eosinophilic keratinocytes (cuticular cells) [15]. In some cases, poromas may display a clear cell change with small nuclei surrounded by a pale cytoplasm (clear cells). Poroid cells are smaller than those in the contiguous epidermis and tend to arrange themselves in cords and broad columns extending downward from the normal epidermis [29]. Some atypical features of malignant tumors can be observed in poroma, including a variable number of mitosing cells, highly vascularized stroma, and necrosis en masse [3,15].

The aggregates of poroid and cuticular cells in a poroma may show ductal or tubular formations; however, the degree of ductal differentiation may vary between each type [1]. Some poromas may have a multitude of ductal foci, whereas others may be more difficult to find [1]. In the latter case, immunostaining with carcinoembryonic antigen (CEA) can highlight the presence of both eccrine and apocrine ducts and thus aid in identification [15]. If the poroma displays more tubular foci lined by columnar cells with holocrine secretions, it is highly suggestive of an apocrine etiology [1].

**Figure 3:**
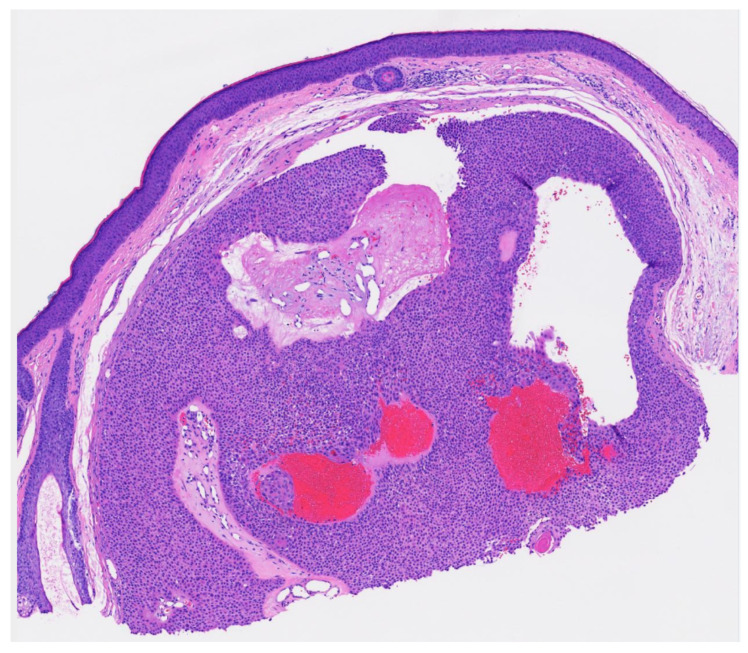
Well circumscribed, dermal-based, solid and cystic tumor with no connection to the overlying epidermis (H & E, 2×).

**Figure 4:**
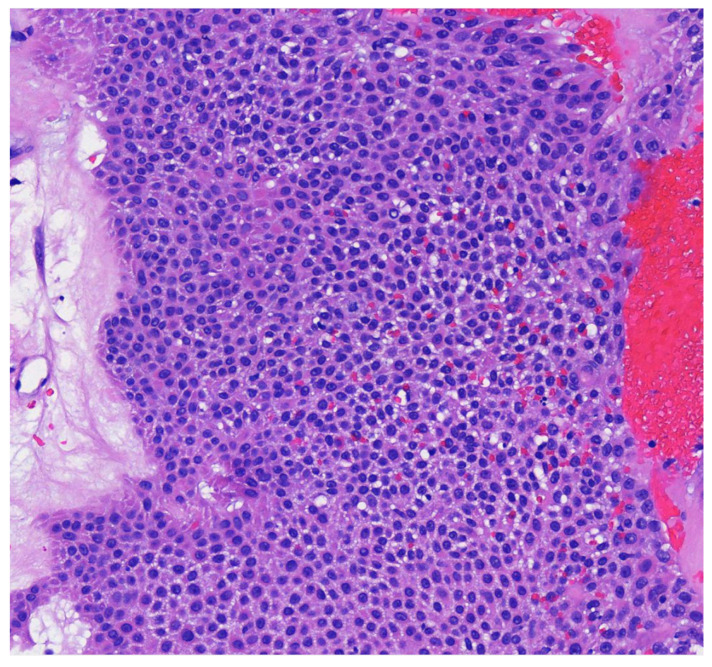
Higher magnification reveals small poriod cells with round to oval nuclei and scant cytoplasm. Ductal lumen formation is present (H & E, 10×).

**Figure 5:**
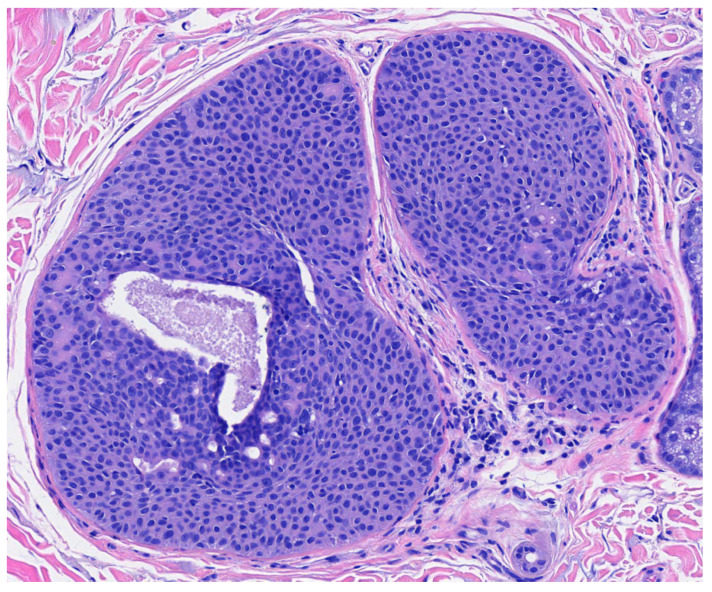
Residual DDT on punch excision (from the transected spiecimen in Figure 3) (H & E, 10×).

Poroma variants are differentiated based on the predominant cell type present and the degree of epidermal/dermal involvement [2]. However, multiple variants can exist within the same lesion. DDTs are primarily confined to the superficial dermis and are composed of small solid and cystic nodular aggregates of poroid, cuticular, and clear cells (Figure 3, Figure 4 and Figure 5) [2]. Eccrine poromas are also composed of all three cell types, but are primarily located in the epidermis and superficial dermis. Hidroacanthoma simplex is mainly composed of poroid cells, less cuticular cells, and no clear cells [2]. It is confined to the epidermis. Poroid hidradenoma contains a mixture of all three cell types and is also confined to the dermis [2]. In contrast to DDT, poroid hidradenomas have large aggregates of solid and cystic components and extend deeper into the reticular dermis and even subcutis [2].

The histopathologic diagnosis of DDT is rare due, in part, to the fact that some regard DDTs as poromas with a limited epidermal connection that has not been identified on the histologic sections examined or as hidradenomas that are smaller and more superficial [30]. As an example, Figure 3 may be regarded by some as representing a hidradenoma. This controversy in the histopathologic classification of poroid neoplasms without clear-cut evidence for the clinical significance of distinguishing these lesions from each other has given rise to the thinking that the poroma classification scheme may just be a matter of semantics [19]. This has led to some pathologists diagnosing these lesions under the overarching term as “acrospiroma” only without further classification.

## 8. Management

Superficial lesions may be treated with shaving or electrosurgical destruction [15]. Deeper lesions may be treated with simple excision [15]. As poromas are benign adnexal lesions, treatment is curative.

## 9. Complications

Malignant transformation risk is minimal; however, poromas may rarely evolve into porocarcinomas. The frequency of porocarcinoma is approximately 0.005–0.01% of all skin cancers [31]. It may present similarly to a benign poroma, as a firm, asymptomatic nodule, but it is typically more exophytic and ulcerative [1]. In the setting of a pre-existing poroma, malignant transformation may be identified clinically by recurrence, spontaneous bleeding, ulceration, sudden itching, pain, or rapid growth [32].

## 10. Conclusions

Poromas are a benign group of tumors derived from cells of the terminal eccrine or apocrine sweat gland duct with four variants: DDT, eccrine poroma, hidroacanthoma simplex, and poroid hidradenoma. DDT is the rarest and is differentiated from the other variants by its cellular composition, as well as its location in the dermis and small foci of solid or cystic components. However, these features may not always be clear cut and can overlap with other variants. Futhermore, all poroma variants can mimic many other dermatologic conditions. Therefore, it is essential to have a complete understanding of poromas to ensure proper diagnosis and treatment.

## Figures and Tables

**Table 1:** Differential diagnosis of sweat gland tumors with a focus on benign tumors with the exception of porocarcinoma.

Differiential Diagnosis of Sweat Gland Tumors [1,6,16,17,18,19,20,21,22,23,24,25,26]					
Condition	Clinical Features	Location	Histological Features	Immunophenotype	Molecular Features
Dermal duct tumor	Dome-shaped papules, plaques, or nodules with color ranging from skin-toned to pink, red, white, or blue. The surface may be smooth, verrucous, or ulcerated.	Primarily dermis	Composed of small solid and cystic nodular aggregates of poroid, cuticular, and clear cells. Ducts, small cystic spaces, and peritumoral clefting is common. Lack prominent cytologic atypia.	Positive for AE1/AE3; ductal structures highlighted by EMA and CEA	YAP1 fusions (YAP1-NUTM1 and YAP1-MAML2)
Poroid hidradenoma		Primarily dermis. Epidermal connections may be present.	Deep-seated solid and cystic nodules composed of bland poroid cells. Areas of biphasic stroma with areas of loose myxoid and of hyaline appearance are common. Ducts are present. Lack prominent cytologic atypia.	Positive for AE1/AE3; ductal structures highlighted by EMA and CEA	YAP1 fusions (YAP1-NUTM1 and YAP1-MAML2)
Hidroacanthoma simplex		Epidermis	Mainly composed of well-circumscribed nests of poroid cells with a few ductal structures. Lack prominent cytologic atypia.	Positive for AE1/AE3; may not demonstrate CEA and EMA positive ductal structures.	YAP1 fusions (YAP1-NUTM1 and YAP1-MAML2)
Eccrine poroma		Epidermis	Composed of cords and broad columns of poriod, cuticular, and clear cells. Ducts and small cysts may be present. Stroma is vascular. Lack prominent cytologic atypia.	Positive for keratins; ductal structures highlighted by EMA and CEA.	YAP1 fusions (YAP1-NUTM1 and YAP1-MAML2)
Porocarcinoma	May be ulcerative or exophytic and demonstrate rapid growth	Epidermis or dermis	Similar to poroma but with invasion, cytologic atypia, numerous mitotic figures, and necrosis.	Positive for AE1/AE3, CK5/6, and p63; ductal structures positive for EMA and CEA.	YAP1 fusions (YAP1-NUTM1 and YAP1-MAML2)
Cylindroma	Solitary papule or nodule	Primarily dermis	Many clusters of small, irregularly shaped aggregations of basaloid cells closely opposed to one another in a jigsaw pattern. Lobules are composed of small basaloid cells and larger pale cells. Hyalinized basement membrane material surrounds the clusters. Focal ductal lumen formation.	Positive for CK6, CK7, CK19, EMA. Basaloid myoepithelial cells positive for SMA, calponin, and S100. Ducts highlighted by CEA and EMA.	CYLD mutations
Spiroadenoma	Solitary papule or nodule, typically ranging from 1 to 3 cm in size	Primarily dermis	One or few large, nodule clusters of small, irregularly shaped aggregations of small basaloid cells and large polygonal cells. Hyalinized basement membrane material surrounds the clusters, forming small circular collections between cells within individual clusters in a trabecular pattern. Intratumoral lymphocytes are present.	Positive for p63, D240, CK7. Often positive for SOX10 and CD117. Myoepithelial cells positive for S100, SMA. Ducts highlighted by CEA and EMA.	ALPK1, CYLD mutations
Hidradenoma	Solitary nodule, typically ranging from 1 to 3 cm in size	Dermis. Epidermal connections occur in 25% of cases.	Deep-seated solid and cystic nodules composed of bland clear, squamoid, basaloid, and mucinous cells. Areas of biphasic stroma with areas of loose myxoid and of hyaline appearance are common. Ducts are present. Lack prominent cytologic atypia.	Positive for AE1/AE3, p40, and p63; ductal structures highlighted by EMA and CEA.	MECT1-MAML2 gene fusion
Hidradenoma papilliferum	Solitary nodule typically ranging from 1 to 3 cm in size, occurs in female genital region	Dermis	Well circumscribed, glands and papillary structures in a maze-like pattern composed of an inner layer of columnar secretory cells with apocrine differentiation and outer layer of cuboidal myoepithelial cells.	CK7, EMA, CEA, GCDFP-15, ER, PR, androgen receptor.	PIK3CA and AKT1 mutations
Syringoma	Multiple 1–4 mm, firm papules	Superficial dermis	Small comma/tadpole-shaped nests and cords of eosinophilic to clear cells with central ducts surrounded by a sclerotic stroma.	CEA, EMA, CK5.	PIK3CA and AKT1 mutations
Syringofibroadenoma	Slow growing, solitary, flesh-colored papules, nodules, or plaques	Superficial dermis	Thin, interconnecting strands of basaloid monomorphous cuboidal cells in a lattice pattern extending from the basal layer of epidermis into dermis.	Luminal cells highlighted by EMA and CEA.	N/A
Syringocystadenoma papilliferum	Firm nodule or plaque most often in head/neck region. May be verrucous. Typically affects younger patients.	Primarily epidermis with extension to superficial dermis	Cystic invaginations of the infundibular epithelium projecting into the dermis, covered by a double cell layer (inner columnar layer with decapitation secretion and outer cuboidal layer with papillary projections. Characteristic plasma cell infiltrate in stroma. True papillary structures more common.	AE1/AE3, EMA, CEA. Myoepithelial layer: CK5/6, SMA, p63.	PTCH, RAS, BRAF, p16 mutations
Cutaneous mixed tumor (chondroid syringoma)	Slow growing firm, painless nodule	Deep dermal to subcutis	Biphasic with both epithelial and stromal components. Stroma is myxoid with cartilaginous metaplasia.	Inner epithelial layer: EMA, CEA, GCDFP-15, actin, CK Outer myoepithelial layer/stromal component: S100, SOX10, NSE, GFAP, SMA, calponin, p63. Variable SMA, GFAP p53 positivity of varying intensity.	PLAG1 or EWSR1 gene rearrangements
Tubular/papillary adenoma	Smooth/irregular well-defined nodule	Deep dermal to subcutis	Lobular pattern of dermal and subcutaneous tubular apocrine structures encased in fibrous/hyalinized stroma. Pseudopapillae are common.	GCDFP-15, CK7. Luminal columnar cells: EMA, CAM5.2, CEA. Outer Tubules: SMA. Myoepithelial Layer: S100.	BRAF and KRAS mutations

## Data Availability

Not applicable.
